# Local Inflammation But Not Kidney Cell Infection Associated with High *APOL1* Expression in COVID-Associated Nephropathy

**DOI:** 10.34067/KID.0000000000000290

**Published:** 2023-11-06

**Authors:** Jane K. Nguyen, Zhenzhen Wu, Jose Agudelo, Leal C. Herlitz, Aaron W. Miller, Leslie A. Bruggeman

**Affiliations:** 1 Robert J. Tomsich Pathology and Laboratory Medicine Institute, Cleveland Clinic, Cleveland, Ohio; 2 Department of Inflammation and Immunity, Cleveland Clinic, Cleveland, Ohio; 3 Department of Cardiovascular and Metabolic Sciences, Cleveland Clinic, Cleveland, Ohio; 4 Department of Urology, Cleveland Clinic, Cleveland, Ohio; 5 Department of Kidney Medicine, Cleveland Clinic, Cleveland, Ohio

**Keywords:** apolipoprotein L1 (APOL1), collapsing FSGS, COVID-19, gene expression, podocyte

## Abstract

- In coronavirus disease-19 biopsies, detection of severe acute respiratory syndrome coronavirus 2 was rare with no evidence of viral replication, whereas autopsy tissue failed quality control.
- In patients with FSGS, apolipoprotein L1 expression differed by degree of immune cell infiltrates, with some podocytes exhibiting up to 18-fold higher expression.
- In COVAN, the predicted high induction of apolipoprotein L1 expression occurs in a pattern consistent with the stochastic nature of FSGS pathology.

## Introduction

The role of severe acute respiratory syndrome coronavirus 2 (SARS-CoV-2) infection in the kidney complications associated with coronavirus disease (COVID) is not fully understood. Although AKI is a frequent complication in hospitalized patients with pneumonia, new-onset or rapidly escalating proteinuria characterized pathologically as a collapsing FSGS is also common. This presentation of FSGS is associated with apolipoprotein L1 (*APOL1*) risk genotypes and has been labeled COVID-associated nephropathy (COVAN) for its resemblance to another viral-induced FSGS, HIV-associated nephropathy (HIVAN). Unlike HIVAN, it remains unclear whether there is a direct role for kidney infection versus an indirect role of antiviral immune responses in pathogenesis. Although several publications have reported evidence of SARS-CoV-2 in kidney tissue, viral RNA or protein detection was infrequent or absent in most cohorts, and if detected, both the number of infected kidney cells and the viral RNA copy number were low (reviewed in refs. 1 and 2).

The proposed mechanism for glomerular injury associated with *APOL1* risk alleles requires secondary triggering events that ultimately induce very high levels of *APOL1* expression causing cytotoxicity.^3^ As APOL1 is expressed in podocytes, this cytotoxicity would result in podocyte depletion, a precipitating event in the formation of segmental scars.^4,5^ Prior studies quantifying glomerular *APOL1* expression in human biopsies, however, have detected no difference or only modestly higher levels of *APOL1* expression in glomeruli of high-risk patients or those with kidney disease.^6–8^ Thus, the proposed high level of *APOL1* expression has not been observed in a naturally occurring APOL1 nephropathy. This high level of *APOL1* expression would be transient because the outcome is cell death, making it difficult to capture this event by examining single–time point biopsies.

Analysis of COVAN samples may be useful in determining the initiating events in an APOL1-dependent FSGS because there is a short time frame from infection and symptom onset to the development of proteinuria and biopsy. This may facilitate observations of key pathogenic events associated with infection, immune responses, and *APOL1* gene expression.

## Methods

Kidney specimens were collected from SARS-CoV-2–positive patients from May 2020 to February 2022 from in-patients undergoing kidney biopsy for diagnostic purposes and from autopsies consented for submission to the local COVID biobank. Study approval and consent waiver were obtained from the Cleveland Clinic institutional review board and adhere to the Declaration of Helsinki. Race/ethnicity was curated as required by the study funder and was self-identified with the following selections available: Asian, Black, Hispanic, multiracial, and White. Pathological diagnosis and scoring were performed by two renal pathologists using standard definitions. Podocytopathy was defined as primary podocyte injury exhibiting diffuse podocyte foot process effacement (by electron microscopy) without evidence of sclerotic changes typical of FSGS. Immune cell infiltrate scoring focused on the tubulointerstitium and used a semiquantitative scale with additional details in Supplemental Methods.

Patient DNA was genotyped for *APOL1* polymorphisms using a TaqMan allele discrimination assay for the G1 allele (rs73885319) and G2 allele (rs71785313). Tissues were examined for viral and host proteins or nucleic acids using formalin-fixed, paraffin-embedded samples. Protein detection by immunofluorescence microscopy used antibodies against SARS-CoV-2 capsid (spike), APOL1, ACE2, CD3, and CD68. RNA detection used the ACDBio RNAscope 2.5 High-Definition RNA *in situ* hybridization kit, either as singleplex or duplex chromogenic manual assays. RNA detection probes included human *APOL1*, *ACE2*, *NPHS1*, *AQP1*, and three probes to detect both sense and antisense SARS-CoV-2 RNA, which detected either genomic RNA or transcripts created during viral replication. A reagent list and details on these methods are provided in the Supplemental Methods.

The ACDBio *in situ* method is a single-molecule detection system that permits quantification of RNA transcripts along with visualization of the tissue structure. Quantification was performed using QuPath from high-resolution images as recommended by ACDBio (Supplemental Methods). Whole-glomerular scoring and single-cell scoring of gene expression were compared using *t* test or ANOVA, with *P* < 0.05 considered significant. Bonferroni *post hoc* test was used for multiple comparisons. Data are presented as mean±SEM, and *P* values are provided in the figure legends.

## Results

The study consisted of 56 patients hospitalized for COVID who were either biopsied for declining renal function (*n*=26) or died of respiratory failure and were autopsied (*n*=30). The characteristics of the autopsy cohort were consistent with prior reports of individuals dying of COVID-19 as being older, obese, and with multiple long-standing comorbidities (Table 1). In the biopsy cohort (Supplemental Table 1), 31% had a preexisting clinical diagnosis of CKD with an additional three patients who received kidney transplants. The most common diagnoses on biopsies obtained during the COVID admission were FSGS (54% collapsing, 11% non-collapsing or not otherwise specified, nos) and podocytopathy (27%) and are consistent with findings in other studies describing COVAN.^2^ The remaining diagnoses (8%) were transplant grafts diagnosed with acute rejection. *APOL1* genotyping identified an enrichment of *APOL1* risk alleles in the biopsy cohort, with all patients carrying at least one *APOL1* risk allele and 65% with an *APOL1* high risk genotype of two *APOL1* risk alleles (Table 2). There were some cases of collapsing FSGS in individuals with *APOL1* low-risk genotypes, which also has been observed in HIVAN, indicating that there are likely other risk factors of collapsing FSGS. Of the Black patients in the autopsy cohort, the prevalence of *APOL1* high risk genotypes (19%) was closer to the population frequency in the local Black community, but 69% carried one *APOL1* risk allele, which is higher than would be expected.

**Table 1:** Cohort demographics and clinical characteristics

Characteristic	Biopsy (*n*=26)	IQR	Autopsy (*n*=30)	IQR	*P* Value
Sex, female, % (*n*)	57.7 (15)		50.0 (15)		n.s.
Age, yr, median	52.5	45–65	61	52–69	0.05
**Race or ethnicity, % (*n*)**					
Black	76.9 (20)		53.3 (16)		
Hispanic	0		6.7 (2)		
Multiracial	0		3.3 (1)		
White	15.4 (4)		36.6 (11)		
Unknown	7.7 (2)		0		
**Comorbidities, % (*n*)**					
Hypertension	61.5 (16)		70.0 (21)		n.s.
Diabetes	38.5 (10)		36.6 (11)		n.s.
Lung disease	3.8 (1)		43.3 (13)		<0.001
Cardiovascular disease	3.8 (1)		43.3 (13)		<0.001
Cancer	n.a.		13.3 (9)		
Prior or current smoker	n.a.		46.6 (14)		
Body mass index, median	n.a.		33.8 kg/m^2^		
Length of disease course, mean	n.a.		32 d		

IQR, interquartile range; na., not available; ns., not significant.

**Table 2:** *APOL1* genotype and risk allele frequencies in Black patients

Cohort	Genotype, % (*n*)			Allele Frequency		
	0 Risk Alleles	1 Risk Allele	2 Risk Alleles	G0	G1	G2
Biopsy cohort^a^ (*n*=20)	0 (0)	35 (7)	65 (13)	0.17	0.58	0.25
Autopsy cohort (*n*=16)	12 (2)	69 (11)	19 (3)	0.47	0.34	0.19
Cleveland population^b^ (*n*=677)	42 (285)	44 (295)	14 (97)	0.64	0.25	0.11

a Genotype for transplant cases are of the graft (donor).

b Originally reported by Miller *et al*.^9^

**Figure 1:**
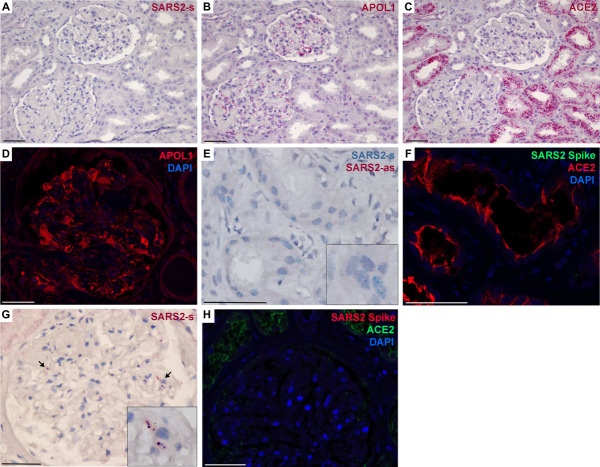
**SARS-CoV-2 was rarely detected in human kidney tissue.** Representative images of kidney biopsy and autopsy tissue for *ACE2*, *APOL1*, and SARS2 sense (s) and antisense (as) RNA by *in situ* hybridization and ACE2, APOL1 and the viral capsid spike protein by immunofluorescence. (A–C) Serial sections of a kidney biopsy that was negative for SARS2 RNA (A), whereas RNA for *APOL1* (B) was evident in podocytes, immune cells, and glomerular and peritubular endothelia, and RNA for *ACE2* (C) was abundant in proximal tubules. (D) The detected *APOL1* RNA in glomeruli also reflected expression of APOL1 protein by immunofluorescence. (E and F) A single kidney biopsy (one of 26) had a region of SARS2-s RNA positivity in tubules that could not be ruled out as artifact; however, this region was negative for SARS2-as RNA and the viral spike protein (F), indicating a lack of viral replication or intact viral particles. (F) Region of positivity shown in (E) was negative for SARS2 spike protein, although ACE2 was abundant. (G) A single kidney autopsy sample (one of 30) had rare evidence of SARS2-s positivity in glomeruli (arrows, magnification in inset). (H) Autopsy specimen shown in (G) was negative for the SARS2 spike protein. Scale bar=50 *μ*m. APOL1, apolipoprotein L1; SARS-CoV-2, severe acute respiratory syndrome coronavirus 2.

SARS-CoV-2 infection of kidney tissue was examined for the presence of viral RNA (by *in situ* hybridization) and for viral proteins (by immunofluorescence). Owing to tissue collection and processing variables, each specimen was evaluated with control probes (Supplemental Figure 1) and with control tissues (Supplemental Figure 2) to verify that tissues were adequately preserved for RNA analyses (identify false negatives) and to verify that detections were not artifacts (identify false positives). All autopsy samples failed these quality controls, with many exhibiting false-positive signals, and were excluded from quantification studies. Overall, the detection of SARS-CoV-2 RNA and protein in kidney tissue was rare, with only one of 26 biopsy and one of 30 autopsy cases generating signals that could not be discounted as artifact (Figure 1). In both of these positive cases, only SARS-CoV-2 genomic RNA was detected and there was no detection of SARS-CoV-2 replication transcripts (Figure 1E) or the viral capsid (spike) protein (Figure 1, F and H). Therefore, in these two positive cases, there was no evidence for viral replication or the presence of intact viral particles. Even in tubules with abundant ACE2 expression (the cell surface docking receptor for viral entry), there was little convincing evidence of viral RNAs or proteins in ACE2-positive cells (Figure 1, C, F, and H).

**Figure 2:**
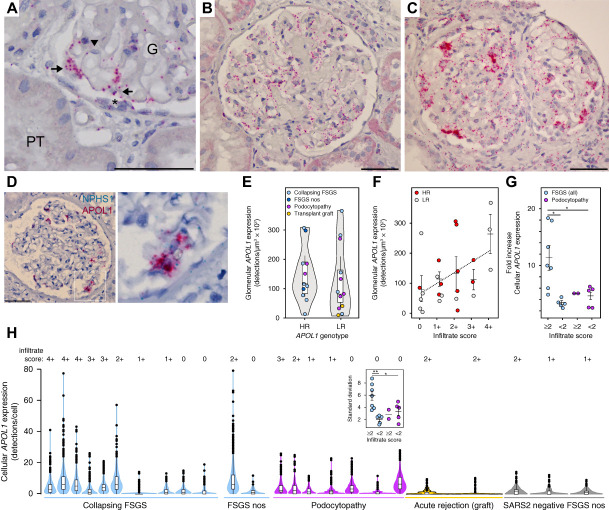
***APOL1* expression was very high in some podocytes in FSGS patients with dense immune cell infiltrates**. RNA *in situ* hybridization for detection and quantification of *APOL1* expression at both the level of whole glomerulus and as individual cells. (A) Representative image of the single-molecule detection of *APOL1* expression in podocytes (arrows) and endothelial cells (arrowhead) and in some parietal cells (*) at tuft adhesions. (B) *APOL1* expression in a patient with FSGS with 1+ infiltrates with a uniform, low-level expression of *APOL1* in podocytes and endothelial cells. (C) *APOL1* expression in a patient with FSGS with 4+ infiltrates showing a fraction of glomerular cells with very high *APOL1* expression. (D) Duplex *in situ* hybridization for *APOL1* and *NPHS1* (Nephrin) indicating that the high expressing glomerular cells were likely podocytes. (E) Violin plot of quantification of *APOL1* glomerular expression means by *APOL1* genotype *APOL1* expression trended higher in high risk genotype patients, but this was not significant (by *t* test). (F) Glomerular *APOL1* expression ordered by degree of immune cell infiltrates with *APOL1* high risk and low risk genotype patients denoted by color coding. FSGS nos, FSGS not otherwise specified. *APOL1* expression trended higher with the higher infiltrate scores (linear regression line *r*^2^=0.2, *P* = 0.17). (G and H) *APOL1* expression quantified at the cellular level. (G) Fold increase in *APOL1* expression of the highest expressing cells compared with expression means. Patients with FSGS with infiltrate scores ≥2 were significantly higher than patients with FSGS with less cellular inflammation or in the other diagnoses (by ANOVA, *P* = 0.0065; *post hoc* test for multiple comparisons *P < 0.05). (H) Violin plots of single-cell *APOL1* expression for each patient ordered by descending infiltrate scores and grouped by kidney diagnosis showing the high-expressing cells outside 95% confidence intervals (outliers shown as dots). SARS2-negative FSGS patients used as technical controls shown for comparison. Inset box compares standard deviations, with significant differences in the patients with FSGS with infiltrate scores ≥2 (by ANOVA, *P* = 0.006; *post hoc* test for multiple comparisons *P ≤ 0.05, **P < 0.01). All box plots show mean line, interquartile range boxes, and 95% confidence interval whiskers. Scale bar=50 *μ*m. G, glomerulus; HR, high-risk; LR, low-risk; PT, proximal tubule.

*APOL1* expression patterns in the biopsy cases also were examined by RNA *in situ* hybridization (Figure 1B), and APOL1 protein was confirmed by immunofluorescence (Figure 1D). *APOL1* expression in glomeruli was evident in podocytes, endothelia, and occasional parietal cells (Figure 2A), with some patients exhibiting a uniform, lower level of expression (Figure 2B), whereas others had sporadic cells with very high *APOL1* expression (Figure 2C). These high-expressing cells also expressed Nephrin (*NPHS1*), a marker of podocytes (Figure 2D). When *APOL1* expression was compared at the level of whole glomeruli, *APOL1* levels trended higher in patients with high risk genotypes compared with low-risk genotypes (Figure 2E). This glomerular quantification is equivalent to experiments quantifying *APOL1* expression using bulk RNA sequencing or microarrays from isolated glomeruli, and this modest increase in *APOL1* expression is consistent with prior studies examining glomerular RNA expression data.^6–8^ In addition, glomerular *APOL1* expression levels trended higher with greater degrees of tissue inflammation using immune cell infiltrate scores, but did not differ by *APOL1* genotype (Figure 2F). *APOL1* gene expression is inducible with some immune mediators and cytokines; however, there is currently no evidence indicating *APOL1* gene expression is regulated differently in the risk variants. Infiltrate scores also were significantly higher in patients with FSGS versus podocytopathies (mean score 2.1 versus 0.9, *P* = 0.037 by *t* test).

The modestly higher level of *APOL1* expression in high risk patients does not fit the proposed mechanism of APOL1-mediated injury, which is believed to require very high levels of *APOL1* expression to trigger cytotoxicity. However, whole-glomerular comparisons represent an average of all expressing cells in which the presence of outliers would be minimized. Examination of *APOL1* expression at the single-cell level (Figure 2, G and H) identified patients with FSGS with a fraction of cells with very high levels of expression outside 95% confidence intervals, which were significant in patients with FSGS with infiltrate scores ≥2 (Figure 2H). These high-expressing cells were up to 18-fold higher than expression means and significantly different than patients with FSGS with less severe tissue inflammation or podocytopathy diagnosis (Figure 2G). In the biopsies, these immune infiltrates in both glomeruli and tubulointerstitium were predominately CD68-positive cells (Supplemental Figure 3), a marker of macrophages, a finding also observed in HIVAN.^10^

## Discussion

By examining patients with COVAN, this is the first clear evidence in human FSGS for the predicted extreme, high levels of *APOL1* expression needed to cause cytotoxicity. The stochastic appearance of these high-expressing cells fits both the current model of APOL1-driven pathogenesis and also the classic pattern of segmental injury that defines FSGS pathology. However, we failed to find convincing evidence of SARS-CoV-2 infection in any renal parenchymal cell type. Unlike HIV-1 and HIVAN, coronaviruses are not integrating viruses, and infected cells do not retain evidence of infection once the infection clears, making the timing of the biopsy critical in ascertaining whether infection was a required part of pathogenesis. It is possible that the transient nature of SARS-CoV-2 infection may underlie the inconsistency in the findings of kidney cell infection. Prior studies with the best evidence of kidney cell infection used autopsy tissue. However, autopsy tissues in our study failed quality control and were compromised by *postmortem* bacterial proliferation or translocation creating false-positive signals (Supplemental Figures 1 and 2).

Our study also failed to find any evidence of viral replication in the kidney and only detected the presence of genomic RNA, the most abundant RNA in SARS-CoV-2 infection and the RNA quantified in circulation. Several groups have successfully infected kidney cells or kidney organoids in vitro (reviewed in ref. 11); however, these experiments circumvented the important issue of whether the kidney is ever exposed to infectious viral particles in vivo. As recently reviewed,^12^ there is little evidence for viremia in SARS-CoV-2 infection. In addition, transplantation of kidneys and other extrapulmonary organs using SARS-CoV-2–positive donors did not transmit virus to the recipient, further supporting the concept kidneys do not harbor infectious virus.^13^ In studies reporting viremia, what is typically measured in blood is not infectious viral particles but viral RNA (RNAemia). Circulating fragmentary viral RNA or viral proteins as noninfectious viral debris as remnants of the respiratory tract infection may passively arrive in the kidney as part of filtration.^14^ Although not infectious, these viral debris may still be pathogenic, altering cellular metabolism and immune responses.

The only association identified with high *APOL1* expression was the severity of tissue inflammation. Although much has been proposed about the storm of circulating cytokines in COVID, it is possible the amount or types of locally produced cytokines in the tissue microenvironment is the critical factor for the pathogenic induction of *APOL1*. In our study, however, it is unclear whether these immune cell infiltrates caused the high *APOL1* expression or only were a manifestation of an ongoing pathogenic process associated with circulating cytokines, nephrotic syndrome,^15^ or the severity of an underlying kidney disease that may have been exacerbated by COVID. This latter point may explain why some patients with FSGS did not exhibit high *APOL1* expression, because the underlying kidney disease may not have been APOL1 nephropathy. Continued work to understand the connection between innate immune signaling events and *APOL1* gene expression will be important to understand both viral and nonviral triggers for FSGS in the APOL1 nephropathies.

## Supplementary Material

SUPPLEMENTARY MATERIAL

## Data Availability

All data is included in the manuscript and/or supporting information.

## Supplemental Material

Supplemental Methods.

Supplemental Table 1. Details on patients in the biopsy cohort.

Supplemental Figure 1. Assay and tissue validation with control probes and control tissues.

Supplemental Figure 2. Common artifacts (false positives) of the ACDBio RNAscope *in situ* hybridization method using chromogenic stains.

Supplemental Figure 3. Immune cells in autopsy and biopsy kidney tissue.

Supplemental References
