# Supplementary material for: Local Inflammation But Not Kidney Cell Infection Associated with High APOL1 Expression in COVID-Associated Nephropathy
Source: Kidney360. 2023 Nov 6;4(12):1757–62. doi: 10.34067/KID.0000000000000290 (PMC10758510; doi:10.34067/KID.0000000000000290)
Supplement: SUPPLEMENTARY MATERIAL [file kidney360-4-1757-s001.pdf]

## Supplemental Information

Local inflammation but not kidney cell infection associated with high *APOL1* expression in COVID-associated nephropathy

**Methods**

*APOL1* genotyping
Infiltrate scoring
Immunofluorescence
In situ hybridization
Quantification with QuPath
Reagent List

**Tables**

Supplemental Table 1. Details on subjects in biopsy cohort.

**Figures**

Supplemental Figure 1. Assay and tissue validation with control probes and control tissues.

Supplemental Figure 2. Common artifacts (false positives) of the ACDBio RNAscope in situ hybridization method using chromogenic stains.

Supplemental Figure 3. Immune cells in autopsy and biopsy kidney tissue.

**Supplemental References**

**Methods**

*APOL1* genotyping

Genomic DNA extractions were from formalin-fixed, paraffin-embedded kidney sections scraped from glass slides (1-2 sections per extraction, depended on amount of tissue on slide). DNA was extracted using the Qiagen RecoverAll total nucleic acid isolation kit following recommended deparaffinization procedures. Final DNA elution was with DNA/RNA-free, DNase/RNase-free water. DNA concentrations were estimated by optical density using spot samples and 5-10ng of DNA were used per each assay. Genotyping for *APOL1* polymorphisms used the TaqMan allele discrimination assay (Thermo Fisher Scientific) for the G1 allele (rs73885319, assay ID C98253221) and G2 allele (rs71785313, assay ID C102754756). Using the dry down method, DNA was pipetted into 384 reaction plates and air dried overnight. TaqPath ProAmp Master Mix (Thermo Fisher Scientific) was mixed with the appropriate TaqMan assay mix for a final volume of 5µl, which was pipetted into each well (positive and negative controls were included on each plate). Amplification used the QuantStudio5 thermocycler (Applied Biosystems) running the QuantStudio Template and Analysis software and using the pre-programmed genotyping amplification protocol (hot start, followed by 40 cycles of 95°C melt for 15 seconds, 60°C amplification for 1 min).

Infiltrate scoring

Renal biopsies were performed for clinically indicated reasons (proteinuria, acute kidney injury) and pathological evaluations were performed by two renal pathologists using standard definitions. Tubulointerstitial lymphocytic inflammation was scored from H&E or PAS stained sections using a semiquantitative scale (0; negative or trace/rare presence of inflammatory cells; 1+, <20% of the tubulointerstitium affected; 2+, 20-40% of the tubulointerstitium affected; 3+, 41-60% of the tubulointerstitium affected; 4+, >60% of the tubulointerstitium affected). All available tissue was scored, which was primarily cortex, but some sections contained medullary tissue.

Immunofluorescence Microscopy

Protein detection by antibody mediated immunofluorescence microscopy with antigen retrieval has been previously described.[1] Sources and specifications of antibodies used are provided in the attached Reagent List. APOL1 antibody specificity has been examined and validated previously.[2] Formalin-fixed, paraffin-embedded 4µm sections were mounted on Fisher superfrost glass slides and were cleared with xylenes, rehydrated in ethanol and washed in water. Antigen retrieval was in 10 mM trisodium citrate dihydrate 6.0, 0.05% Tween-20 using a pressure cooker,

boiling under pressure for 4.5 min. Slides were cooled to room temperature, rinsed in PBS, and transferred to PBST (PBS with 0.2% Tween-20). Slides were blocked with 5% normal goat serum in PBST for 1 hour, and incubated overnight at 4°C in PBS, 1% normal goat serum, 0.1% Tween-20 with the appropriate dilution of primary antibodies (see Reagent Table). Following incubation, slides were washed with PBST, and incubated with species specific fluorophore-conjugated secondary antibody in PBS for 1 hour at room temperature and washed with PBST. Slides were mounted in antifade mounting media containing DAPI. Fluorescent images (2048 x 2048 pixels, 72 pixels/inch) were captured using Leica LAS-X software on a Leica TS-SP8-AOBS inverted confocal microscope using 405, 488, and 561 lasers at 40X magnification.

In situ hybridization

The RNA in situ hybridization for gene expression was performed using manual kits from ACDBio following kit instructions (See Reagent List for details on kits and probes). Manual tissue pretreatment conditions (target retrieval and protease digestion) for both lung and kidney tissue were 15 minute boiling and 30 minute protease digestion. Samples that failed under these conditions were repeated with modifications to the pretreatments (combinations of boiling times ranging from 10 to 30 minutes, and protease digestion times ranging from 10 to 30 minutes). However, failed samples continued to fail under these optimizing runs and likely represented samples with significant RNA degradation. Other than optimizing tissue pretreatment conditions, there was no deviation from the provided kit protocol. Slides were mounted in EcoMount, dried overnight, and visualized using light microscopy (see below).

The fluorescent in situ hybridization (FISH) procedure to detect bacteria used 5'-labeled Alexa fluor 660 DNA probes (synthesized at Invitrogen) and conditions as previously described.[3] Briefly, deparaffinized tissue sections were incubated with the FISH probe (3ng/μl, see Reagent List) in hybridization buffer (100 mM Tris–HCl pH 7.2, 0.9 M NaCl, and 0.1% SDS, and RNA stabilization solution) using 50 μl/slide, and a cover slip was floated on the tissue section, followed by incubation for 90 minutes at 60ºC. Coverslips were removed and slides were washed with 100 mM of Tris-HCL pH 7.2, 0.9 M NaCl, 0.1 mM SDS for 30 minutes. Slides were mounted in antifade mounting media with DAPI and imaged as described above for immunofluorescence microscopy.

Quantification of gene expression.

ACDBio recommends QuPath,[4] an open source (https://qupath.github.io/) digital pathology and image analysis program, for quantification of single molecule detections. QuPath file and stain definitions were programmed as recommended by ACDBio in their analysis guidelines (https://acdbio.com/qupath-rna-ish-analysis) and parameters used in cell and spot detection are summarized in the adjacent table. Of note, in high expressing cells any signal clusters (i.e., the merging or overlap of single molecule signals) were quantified using the "split by intensity" method. Light micrograph images (TIFF format, 1024 x 1280 pixels, 72 pixels/inch) at 40X magnification were collected on a Nikon Eclipse 55i microscope using Nikon Digital Sight 10 frame capture and NIS Elements software (Nikon). Raw images were imported into QuPath and manually annotated for individual glomeruli (all available in biopsies) or proximal tubules (an average of eight tubules per specimen from three separate 40X fields). Biopsy cores that had less than three glomeruli were excluded from single cell quantifications. Data output for subcellular detections was collected for both the annotated region (used for whole glomeruli scoring) and for each cell within the annotated region (used for single cell scoring). QuPath also provided data for areas and volumes for both annotated regions and cells, with volumes calculated based on a specimen thickness of 4µm.

| QuPath settings | | Glomeruli | Proximal tubule |
|---|---|---|---|
| nucleus parameters | background radius | 40 | 40 |
| | medan fl radius | 0 | 0 |
| | sigma | 6 | 8 |
| | min area | 150 | 450 |
| | max area | 1500 | 2000 |
| | threshold | 0.1 to 1.5 | 0.05 to 0.1 |
| | max backgroud | 2 | 2 |
| | split by shape | yes | yes |
| cell parameters | cell expansion | 75 | 30 |
| | include nucleus | yes | yes |
| | smooth boundries | yes | yes |
| subcellular spot detection | detect threshold | 0.2 | 0.2 |
| | smooth | no | no |
| | split by shape | no | no |
| | split by intensity | yes | yes |
| spot & cluster parameters | expect spot size | 1 | 1 |
| | min spot size | 1 | 1 |
| | max spot size | 200 | 50 |
| | include clusters | yes | yes |

## Reagent list

| Methods | Reagent | Company | Catalog number | application details |
|---|---|---|---|---|
| RNA in situ hybridization | RNAscope 2.5 HD Duplex kit (manual) | ACDBio | 322430 | |
| | RNAscope 2.5 HD RED Assay (manual) | ACDBio | 322350 | |
| | *Hs-APOL1-No-XMm* | ACDBio | 459791 | channel 1 or channel 2 if in duplex assay |
| | *Hs-AQP1* | ACDBio | 421831 | channel 1 or channel 2 if in duplex assay |
| | *Hs-NPHS1* | ACDBio | 416071 | channel 1 or channel 2 if in duplex assay |
| | *Hs-ACE2* | ACDBio | 848151 | channel 1 or channel 2 if in duplex assay |
| | *V-nCoV2019-S* | ACDBio | 848561 | channel 1 or channel 2 if in duplex assay |
| | *V-nCoV2019-S-sense* | ACDBio | 845701 | channel 1 or channel 2 if in duplex assay |
| | *V-nCoV2019-orf1ab-sense* | ACDBio | 859151 | channel 1 or channel 2 if in duplex assay |
| | EcoMount | ACDBio | EM897L | |
| Fluorsecent in situ hybridization | bacterial taxa universal 16s | Invitrogen | EUB388 | GCTGCCTCCCGTAGGAGT |
| Immunofluorescence microscopy | anti-human APOL1 rabbit polyclonal | Sigma | HPA018885 lot 105260 | 1:400 dilution (note: this lot is no longer available for purchase from manufacturer) |
| | anti-human CD3 rabbit monoclonal | Abcam | ab16669 | 1:3000 dilution |
| | anti-human CD68 mouse monclonal | Abcam | ab955 | 1:400 dilution |
| | anti-human ACE2 rabbit monoclonal | Invitrogen | MA5-32307 | 1:100 dilution |
| | anti-SARS-CoV-2 nucleocapsid (spike) mouse monoclonal | Invitrogen | MA1-7404 | 1:200 dilution |
| | Goat anti-rabbit Cy3 | Jackson Labs. | 111-165-144 | 5 µg/ml |
| | Goat anti-mouse Cy3 | Jackson Labs. | 115-165-146 | 5 µg/ml |
| | Goat anti-rabbit Alexafluor 488 | Invitrogen | A11034 | 5 µg/ml |
| | Goat anti-mouse Alexafluor 488 | Invitrogen | A11029 | 5 µg/ml |
| | Slowfade Diamond mountant with DAPI | Invitrogen | S36964 | |

**Supplemental Table 1**: Details on subjects in biopsy cohort.

| sex | age | *APOL1* genotype | biopsy diagnosis | clinical AKI | prior CKD diagnosis | other clinical data | pre-existing diabetes | pre-existing hypertension |
|---|---|---|---|---|---|---|---|---|
| F | 46 | G1G1 | Collapsing FSGS | | yes | | | |
| M | 71 | G2G2 | Collapsing FSGS | yes | yes | | yes | yes |
| F | 47 | G1G1 | Collapsing FSGS | yes | yes | | | yes |
| F | 64 | G1G1 | Collapsing FSGS | yes | | | yes | yes |
| M | 54 | G1G2 | Collapsing FSGS | yes | yes | | | yes |
| M | 66 | G1G2 | Collapsing FSGS | yes | yes | | | yes |
| M | 77 | G0G1 | Collapsing FSGS | yes | | | | yes |
| M | 45 | G0G1 | Collapsing FSGS | yes | | | | yes |
| F | 55 | G0G1 | Collapsing FSGS | yes | | | | yes |
| M | 46 | G2G2 | Collapsing FSGS | yes | | | | |
| F | 77 | G1G1 | Collapsing FSGS | yes | | | | yes |
| M | 39 | G0G0 | Collapsing FSGS | yes | yes | | | |
| M | 45 | G0G1 | Collapsing FSGS | | | | | |
| F | 67 | G0G1* | Collapsing FSGS | yes | yes | transplant recipient | | |
| F | 56 | G1G2 | FSGS nos | yes | | | yes | yes |
| F | 30 | G1G1 | FSGS nos | | | | yes | yes |
| F | 61 | G1G1 | FSGS nos | | | | | yes |
| M | 44 | G0G2 | Podocytopathy | | yes | | | |
| F | 44 | G0G0 | Podocytopathy | | | | yes | |
| F | 51 | G0G0 | Podocytopathy | | | | yes | |
| F | 51 | G1G2 | Podocytopathy | yes | | | yes | yes |
| F | 69 | G0G0 | Podocytopathy | | | | yes | yes |
| F | 60 | G1G1 | Podocytopathy | | | pre-existing lupus | yes | yes |
| M | 21 | G0G0 | Podocytopathy | yes | | | | |
| F | 67 | G0G2* | acute rejection | yes | yes | transplant recipient | yes | yes |
| M | 45 | G0G0* | acute rejection | yes | yes | transplant recipient | | |
| COVID negative controls | | | | | | | | |
| M | 59 | G0G0 | FSGS nos | | yes | | yes | yes |
| M | 53 | G0G0 | FSGS nos | | yes | | | yes |
| M | 53 | G0G0 | FSGS nos | | yes | | | yes |
| M | 32 | G0G0 | IgA nephropathy | | yes | | | |
| F | 65 | G0G0* | normal renal parenchyma (implant biopsy) | | | | | |

* graft/donor genotype

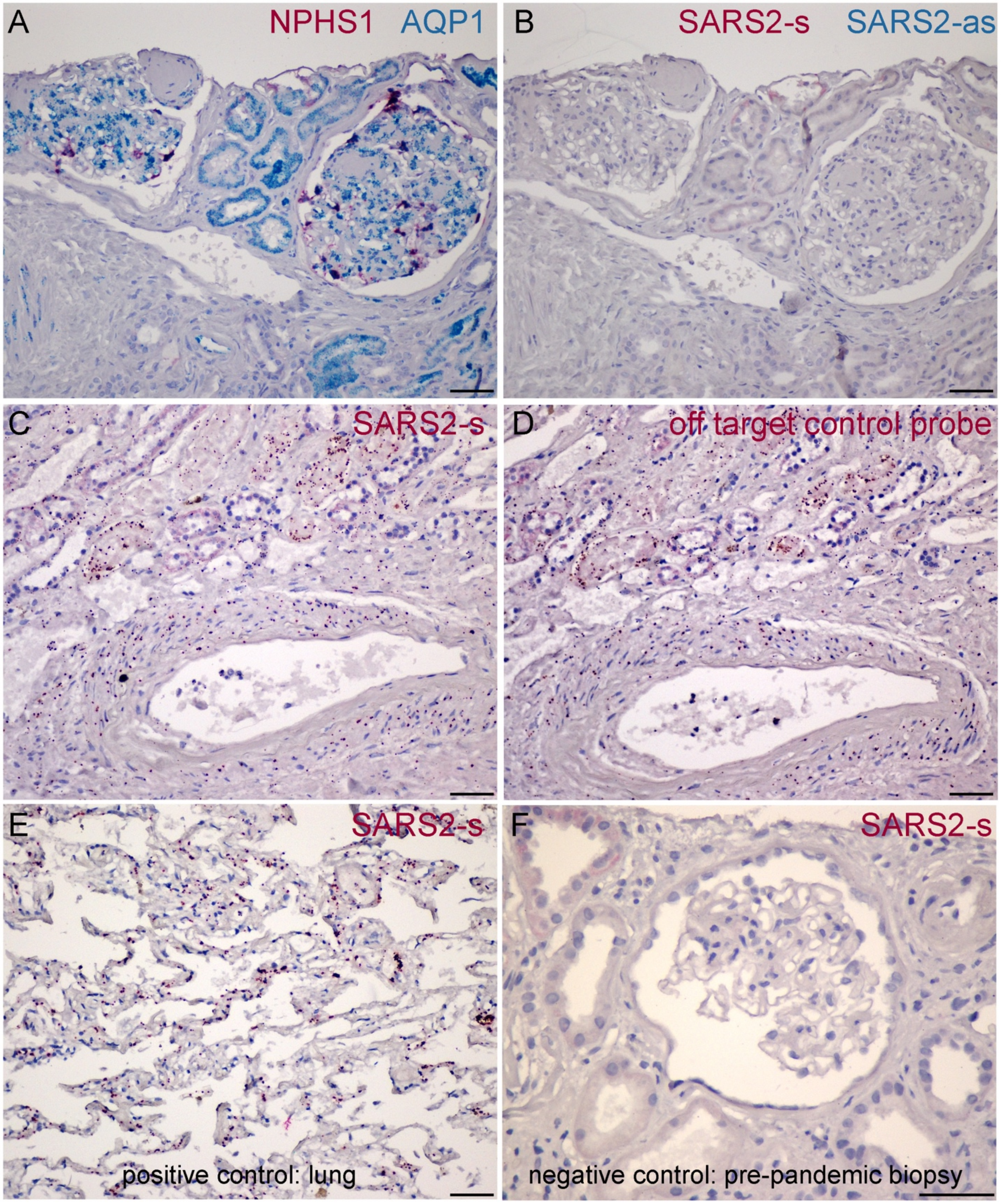


**Supplemental Figure 1. Assay and tissue validation with control probes and control tissues.**

**Supplemental Figure 1.** Representative results using ACDBio RNAscope in situ hybridization manual assay using either one probe (singleplex, fast red chromogen) or two probes (duplex, fast red and fast green chromogens). Slides are counterstained with hematoxylin. **A**. Example of tissue quality control using the RNAscope duplex assay with positive control probes for the genes for nephrin (*NPHS1*, expected signal in podocytes) and aquaporin 1 (*AQP1*, expected signal in proximal tubules and glomerular endothelia). Tissues that failed these two probes were considered to not pass quality control and indicated the tissue handling and storage conditions were not adequate to preserve RNA. This quality control step eliminated potential false negatives. **B**. Serial section to panel A showing absence of SARS-CoV-2 (SARS2) RNA using both a probe to detect viral genomic RNA (sense, s) or viral RNAs generated during active replication (antisense, as). **C, D**. Example of a suspect artifactual positive signal (**C**) verified by comparing with an off target probe (**D**) in serial sections. Probe in panel D should be negative but generated a similar pattern to panel C which was likely bacterial contamination (see supplemental Figure 2). **E**. Lung tissue obtained from COVID autopsy case used as a positive control. **F**. Biopsies from SARS-CoV-2 negative subjects (n=5), including biopsies obtained prior to the pandemic, were used as negative controls. Scale bar = 50μm.

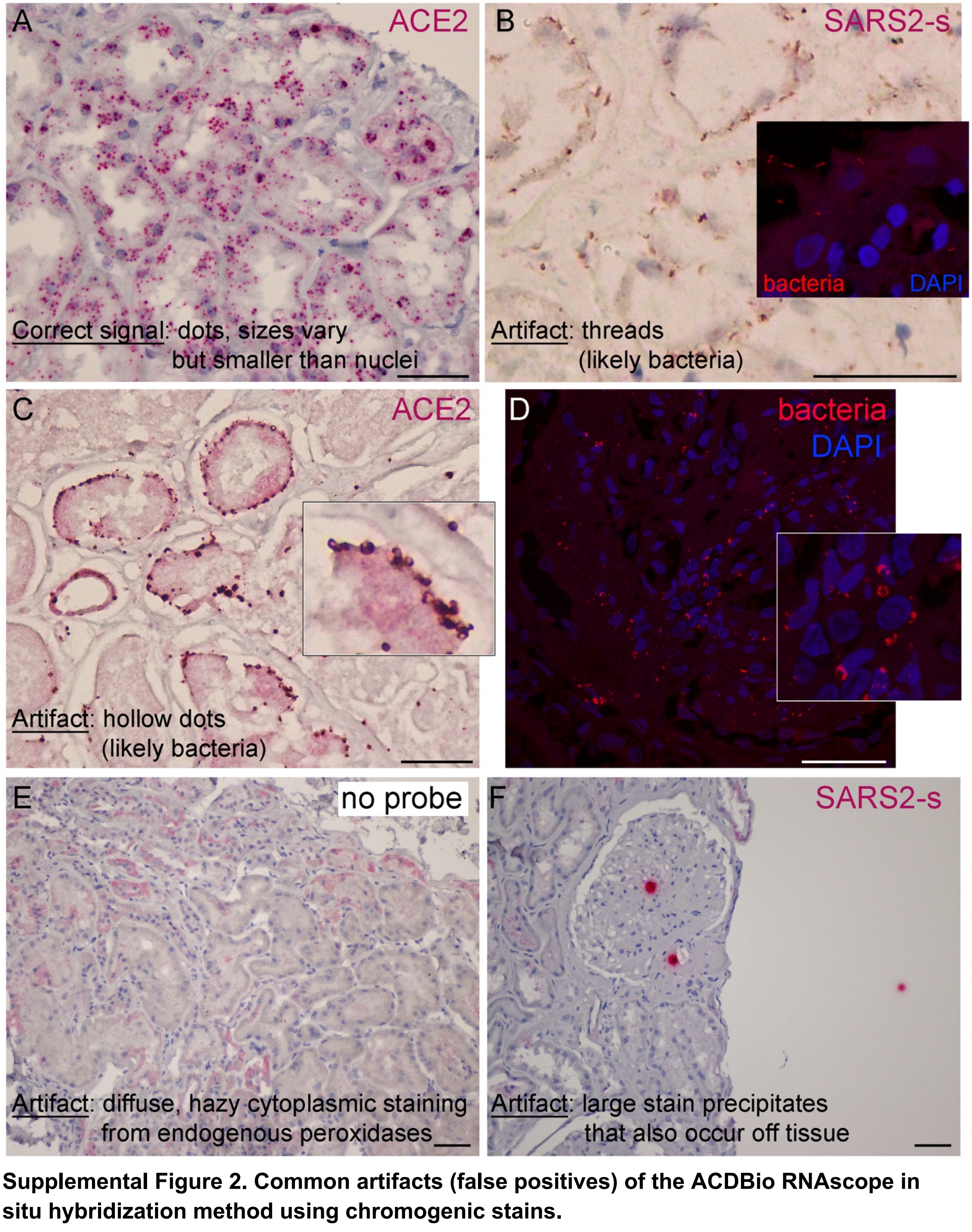


**Supplemental Figure 2. Common artifacts (false positives) of the ACDBio RNAscope in situ hybridization method using chromogenic stains.**

**Supplemental Figure 2.** ACDBio RNAscope in situ hybridization manual assay using one probe (fast red chromogen), counterstained with hematoxylin. **A**. The correct signal from the RNAscope assay is a dot of varying sizes, but dots are smaller than nuclei. They represent single molecule detection and can be quantified using standard image analysis techniques. **B-D**. Bacterial contamination, which was most problematic in the autopsy specimens, generated positive signals that frequently were not dots, but either threads (panel **B**) or hollow dots or curved structures (panel **C**). Specimens that exhibited this pattern were re-examined with an off-target probe (see sFig 1 C, D) or bacterial contamination was confirmed by using a fluorescent in situ hybridization procedures with a universal (pan-bacteria) 16s rRNA probe (panel **D** and panel **C** inset); DAPI as a nuclear stain. **E**. Another common artifact is the bleed-through or inadequate blocking of endogenous peroxidases, resulting in a diffuse or hazy cytoplasmic staining most commonly observed in tubules. This artifact was confirmed/ruled-out by processing additional sections through the complete in situ procedure but excluding the probe and replacing it with probe diluent (no probe control). **F**. Another artifact is the non-specific deposition of stain precipitates, which can be discerned from a *bona fide* RNA detection signal as the precipitates typically form dots larger than the size of nuclei and are also observed off the tissue section. This artifact was confirmed/ruled-out by repeating the assay on additional sections to determine if the signal was reproducible. Scale bar = 50μm.

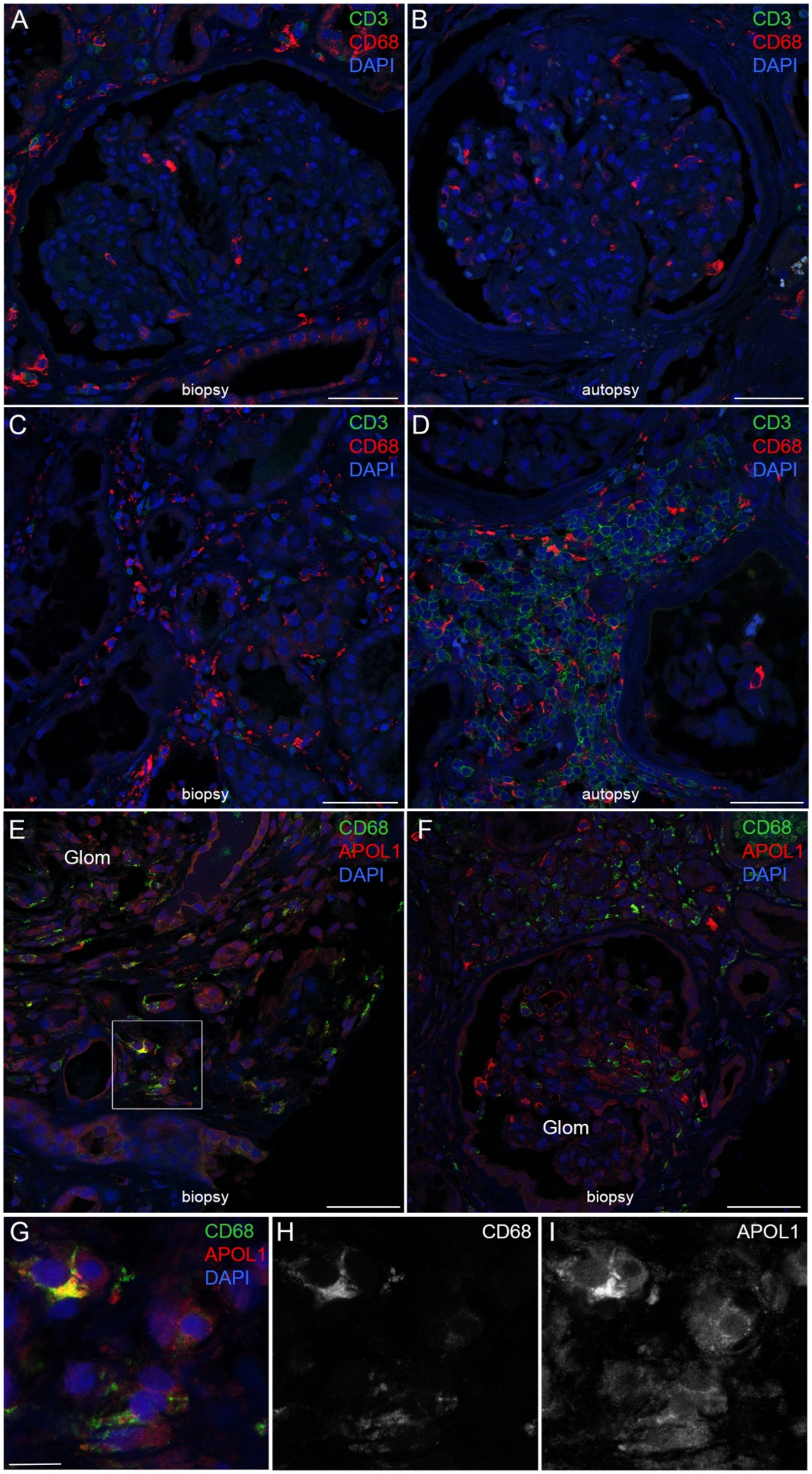


**Supplemental Figure 3. Immune cells in autopsy and biopsy kidney tissue**.

**Supplemental Figure 3. Immune cells in autopsy and biopsy kidney tissue**.

Immunofluorescence for CD3 (pan T cell marker) and CD68 (monocyte/macrophage marker), with DAPI as nuclear stain. **A**, **C**. Biopsies had varied degrees of immune cell infiltrates, but those with immune cell infiltrates were primarily populated by CD68 positive cells with few CD3 positive cells in both glomeruli (A) and tubulointerstitium (C). **B**, **D**. Autopsy specimens also had varying degrees of immune cell infiltrates, but those with infiltrates were populated by both CD3 and CD68 positives cells in both glomeruli (B) and tubulointerstitium (D). **E**-**I**. In biopsies, CD68 positive immune cells expressed apolipoprotein L1 (APOL1). **E, F**. CD68-positive Immune cells in glomeruli (Glom) and interstitium also express APOL1. It has been shown previously that cells of immune lineages including macrophages, NK cells and T cells express *APOL1* and may participate in events that effect outcomes associated with kidney transplantation.[5-8] **G**. Boxed region in panel E at higher magnification with individual fluorescent channels for CD68 and APOL1 shown in panels **H** and **I** respectively as monochromatic images. Panels A-F, scale bar = 50μm. Panels G-I, scale bar = 10μm.

**Supplemental References**


1. Madhavan SM, O'Toole JF, Konieczkowski M, Ganesan S, Bruggeman LA, Sedor JR: APOL1 localization in normal kidney and nondiabetic kidney disease. *J Am Soc Nephrol,* 22**:** 2119-2128, 2011 10.1681/asn.2011010069
2. Blessing NA, Wu Z, Madhavan SM, Choy JW, Chen M, Shin MK, et al.: Lack of APOL1 in proximal tubules of normal human kidneys and proteinuric APOL1 transgenic mouse kidneys. *PLoS One,* 16**:** e0253197, 2021 10.1371/journal.pone.0253197
3. Daims H, Brühl A, Amann R, Schleifer KH, Wagner M: The domain-specific probe EUB338 is insufficient for the detection of all Bacteria: development and evaluation of a more comprehensive probe set. *Syst Appl Microbiol,* 22**:** 434-444, 1999 10.1016/s0723-2020(99)80053-8
4. Bankhead P, Loughrey MB, Fernández JA, Dombrowski Y, McArt DG, Dunne PD, et al.: QuPath: Open source software for digital pathology image analysis. *Scientific Reports,* 7**:** 16878, 2017 10.1038/s41598-017-17204-5
5. Zhang Z, Sun Z, Fu J, Lin Q, Banu K, Chauhan K, et al.: Recipient APOL1 risk alleles associate with death-censored renal allograft survival and rejection episodes. *J Clin Invest,* 131, 2021 10.1172/jci146643
6. Taylor HE, Khatua AK, Popik W: The innate immune factor apolipoprotein L1 restricts HIV-1 infection. *J Virol,* 88**:** 592-603, 2014 10.1128/jvi.02828-13
7. Lee H, Fessler MB, Qu P, Heymann J, Kopp JB: Macrophage polarization in innate immune responses contributing to pathogenesis of chronic kidney disease. *BMC Nephrol,* 21**:** 270, 2020 10.1186/s12882-020-01921-7
8. Ryu JH, Ge M, Merscher S, Rosenberg AZ, Desante M, Roshanravan H, et al.: APOL1 renal risk variants promote cholesterol accumulation in tissues and cultured macrophages from APOL1 transgenic mice. *PLoS One,* 14**:** e0211559, 2019 10.1371/journal.pone.0211559
